# Associations between postnatal cerebral oxygen availability and utilization in very to late preterm infants and neurodevelopmental outcome

**DOI:** 10.1038/s41598-026-35303-0

**Published:** 2026-01-11

**Authors:** Anurudhya Karthikeyan, Thuy Mai Luu, Rasheda Chowdhury, Ramy El-Jalbout, Mi-Suk Kang Dufour, Olivia Beaulieu, Léanne Brabant, Gabriel Côté-Corriveau, Marie-Michèle Gagnon, Mélanie Gagnon, Catherine Bernard, Véronique Belval, Anne-Monique Nuyt, Marie-Noëlle Simard, Mathieu Dehaes

**Affiliations:** 1 Centre de recherche Azrieli du CHU Sainte-Justine, Montreal, Canada; 2 Institute of Biomedical Engineering, Université de Montréal, Montreal, Canada; 3 Department of Pediatrics, CHU Sainte-Justine University Hospital Centre, Université de Montréal, Montreal, Canada; 4 Department of Radiology, Radio-oncology and Nuclear Medicine, Université de Montréal, Montreal, Canada; 5 School of Public Health, University of California, Berkeley, USA; 6 Direction des services multidisciplinaires, de la santé mentale et de la réadaptation, CHU Sainte-Justine University Hospital Centre, Montreal, Canada

**Keywords:** Cerebral oxygen delivery, Cerebral blood flow, Cerebral oxygen metabolism, Frequency-domain near infrared spectroscopy (FDNIRS) and diffuse correlation spectroscopy (DCS), Neurodevelopment, Prematurity, Biomarkers, Medical research, Neurology, Neuroscience

## Abstract

Preterm children are more likely to experience neurodevelopmental challenges than neonates born at term. Early biomarkers of brain development may help clinicians to identify children in need of early interventions. The main objective of this study was to assess the associations between postnatal cerebral oxygen availability and utilization with neurodevelopmental outcome in preterm infants and explore sex-specific differences. This prospective observational cohort included 227 infants born at 29–36 weeks of gestation who underwent bedside optical neuromonitoring around term-equivalent age to measure cerebral oxygen availability (blood flow and delivery [CDO_2i_]) and utilization (metabolism [CMRO_2i_] and extraction fraction [OEF]). Neurodevelopmental outcome was assessed at 2 years corrected age. Linear regression analyses were used to assess the associations between neuromonitoring parameters and neurodevelopmental scores. Increased CDO_2i_, CMRO_2i_ and OEF were associated with higher cognitive and language scores. When stratifying by sex, most of these associations were stronger in males than females. Associations between neuromonitoring parameters and motor scores were observed only in males. These results support the potential role of bedside optical neuromonitoring to provide early biomarkers of brain health and maturation in preterm infants and suggest sex-specific developmental mechanisms, which may indicate higher vulnerability in males.

## Introduction

Preterm birth accounts for approximately 8% of all live births yearly in Canada^1^, with the majority occurring at 29–36 weeks of gestational age (GA). Compared to term children, infants born at 29–36 weeks’ GA are more likely to experience neurodevelopmental challenges^2–6^. Yet, structured neurodevelopmental follow-up is predominantly geared towards extremely preterm infants (< 28 weeks’ GA)^2,7,8^. Identifying preterm infants born at 29–36 weeks’ GA who would also benefit from such neurodevelopmental follow-up is clinically relevant.

Altered brain development in preterm infants has been well described using magnetic resonance imaging (MRI) and is characterized by lower global and regional brain volumes and metabolism^9,10^ as well as disrupted white matter microstructure and functional connectivity^11–16^. Clinically, higher MRI brain abnormality scores have been associated with suboptimal neurobehaviour at term-equivalent age (TEA)^17,18^. Brain volumes and diffusion MRI parameters were also associated with 2-year neurodevelopmental scores^19,20^. Sex-specific differences were previously reported, with preterm males more likely vulnerable^11,21–25^.

MRI biomarkers are valuable, but expensive and challenging to apply at the bedside. Bedside conventional near infrared spectroscopy (NIRS) has been used to measure relative cerebral hemoglobin oxygen saturation at birth in very preterm children; high and low values were associated with neurodevelopmental delay at 2 to 3 years^26–28^. Also, in extremely preterm infants, there was no association between NIRS values at birth and 2-year outcome^29^. The use of NIRS in the clinical care of extremely preterm infants was not associated with a lower incidence of adverse outcome at 36 weeks postmenstrual age (PMA)^30^. Although findings remain inconsistent, conventional NIRS measurements of cerebral hypo- and hyperoxygenation may still have the potential to inform on brain health.

A variant of conventional NIRS is the combination of frequency-domain NIRS (FDNIRS) and diffuse correlation spectroscopy (DCS), which allows for monitoring of cerebral oxygen availability (via indices of blood flow [CBF_i_] and oxygen delivery [CDO_2i_]) and utilization (via oxygen metabolism index [CMRO_2i_] and extraction fraction [OEF]). Previous studies have reported these bedside FDNIRS-DCS parameters in preterm children^31–36^. However, how FDNIRS-DCS parameters measured around TEA are associated with long-term neurodevelopment in preterm infants is unknown.

In this study, we aimed to assess the associations between FDNIRS-DCS parameters measured around TEA and 2-year neurodevelopmental outcomes in children born at 29–36 weeks’ GA. The secondary aim was to assess the associations between PMA and FDNIRS-DCS parameters. Finally, we explored sex-specific differences in these associations. We hypothesized that postnatal FDNIRS-DCS neuromonitoring parameters quantifying cerebral oxygen availability and utilization are associated with neurodevelopment and PMA, and are sex-specific.

## Results

### Population characteristics

A total of 241 infants born between 29 and 36^6/7^ weeks’ GA and admitted to the neonatal intensive care unit (NICU) for at least 48 h after birth were included (882 were consecutively approached). In 10 multiple births, both siblings were recruited: 4 were twin pairs of females, 4 were twin pairs of males and two were mixed sex pairs. From this cohort, 227 underwent bedside optical neuromonitoring at TEA. Table 1 shows demographic, clinical, neuromonitoring, and neurodevelopmental variables of these patients (126 males). Median gestational age at birth was 33 weeks (interquartile range [IQR] 31.6–34.4) and median birth weight was 1780 g (IQR 1480–2175), which was lower in females compared to males. Socioeconomic status was low in 14.9% of the families and maternal education lower or equal to high school was higher in males than females. At TEA, 37% of infants displayed abnormal neurological status, which was higher in males compared to females. Time from birth to discharge was higher in females (21 days, IQR 14–36) than males (17 days, IQR 12–30). Median PMA at the time of neuromonitoring was 39.9 weeks (IQR 37.6–40.6). Median CMRO_2i_ and hemoglobin concentration in the blood (HGB) were higher in males compared to females while absolute cerebral hemoglobin oxygen saturation (SO_2_) lower. Median corrected age at neurodevelopmental assessment was 26 months (IQR 25–26). Neurodevelopmental data were completed for 204 infants (115 males). Mean cognitive, language and motor scores were 98.4 (standard deviation [SD] 13.5), 95.2 (SD 19.9) and 99.4 (SD 11.1), respectively. Males had lower language and motor scores than females.

**Table 1:** Characteristics of study participants.

Characteristics	Full Cohort	Males	Females
	**(n = 227)**	**(n = 126)**	**(n = 101)**
Gestational age, median (IQR), weeks	33 (31.6, 34.4)	33.4 (31.5, 34.6)	33.1 (31.6, 34.4)
Birth weight, median (IQR), g	1780 (1480, 2175)	**1820 (1573**,** 2238)**	**1680 (1430**, 2050)^*^
Birth weight z-score, median (IQR)	−0.26 (−1.16, 0.32)	−0.26 (−1.16, 0.33)	−0.27 (−1.11, 0.3)
Head circumference, median (IQR), cm	29.5 (28, 31)	29.6 (28, 31.5)	29.5 (28, 30.5)
Multiple birth, n (%)	64 (28.2)	31 (24.6)	33 (32.7)
Small for gestational age, n (%)	90 (39.6)	49 (38.9)	41 (40.6)
Intrauterine growth restriction, n (%)	79 (24.8)	44 (34.9)	35 (34.7)
Retinopathy of prematurity, n (%)	7 (3.08)	4 (3.17)	3 (2.97)
Sepsis/meningitis, n (%)	4 (1.76)	4 (3.17)	0 (0)
Intraventricular haemorrhage, n (%)^a^	11 (7.8)	6 (7.89)	5 (7.69)
Periventricular leukomalacia, n (%)^a^	1 (0.7)	1 (1.32)	0 (0)
Bronchopulmonary dysplasia, n (%)	16 (7.05)	8 (6.35)	8 (7.92)
Antenatal corticosteroids, n (%)	157 (69.2)	84 (66.7)	73 (72.3)
Urgent cesarian section, n (%)	101 (71)	54 (42.9)	47 (46.5)
Surfactant administration, n (%)	44 (19.4)	26 (20.6)	18 (17.8)
Invasive mechanical ventilation, n (%)	37 (16.3)	23 (18.3)	14 (13.9)
Non-invasive mechanical ventilation, n (%)	160 (70.5)	84 (66.6)	76 (75.2)
Low socio-economic status, n (%)	32 (14.9)	19 (16)	13 (13.5)
Maternal education ≤ high school, n (%)	40 (18.5)	**29 (24.2)**	**11 (11.5)** ^*^
Abnormal neurological status at TEA, n (%)	82 (38.1)	**54 (45.4)**	**28 (29.2)** ^*^
Neonatal mortality, n (%)	2 (0.88)	2 (1.59)	0 (0)
Time from birth to discharge, median (IQR), days	19 (13, 35.5)	**17 (12**,** 30)**	**21 (14**, 36)^*^
Neuromonitoring at TEA			
Infant state, n (%)	212 (93.4)	115 (91.3)	97 (96)
Awake	46	27	19
Asleep	144	75	69
Both awake and asleep	22	13	9
Time at neuromonitoring (24 h system), median (IQR)	11:20 (10:31, 12:05)	11.20 (10:20, 12:05)	11:18 (10:40, 12:05)
PMA, median (IQR), weeks	39.9 (37.6, 40.6)	39.7 (37.8, 40.4)	40 (37.4, 40.6)
HGB, median (IQR), g/dL	13.3 (10.4, 15.9)	**13.7 (10.9**,** 16.2)**	**12.8 (9.8**, 15.1)^*^
CBF_i_, median (IQR), mm^2^/s × 10^− 5^	0.43 (0.33, 0.51)	0.42 (0.33, 0.53)	0.44 (0.34, 0.5)
CDO_2i_, median (IQR), ml O_2_/dl × mm^2^/s × 10^− 4^	0.72 (0.56, 0.93)	0.75 (0.55, 0.02)	0.71 (0.6, 0.88)
CMRO_2i_, median (IQR), ml O_2_/dl × mm^2^/s × 10^− 4^	0.3 (0.2, 0.42)	**0.35 (0.2**,** 0.45)**	**0.28 (0.2**, 0.37)^*^
OEF, median (IQR), %	42 (30.4, 51.8)	44 (32.4, 52.9)	40 (28.5, 50.8)
SO_2_, median (IQR), %	65.6 (59.5, 75.4)	**64.4 (59.2**,** 74.5)**	**68.7 (60.6**, 77.3)^*^
Neurodevelopment at age 2 years			
Corrected age, median (IQR), months	26 (25, 26)	26 (25, 26)	25 (25, 26)
Completed follow-up, n (%)	204 (90)	115 (91.3)	89 (88)
Cognitive score, mean (SD)	98.4 (13.5)	97 (14.04)	100.3 (12.6)
Language score, mean (SD)	95.2 (19.9)	**92.5 (18.6)**	**98.7 (21.1)** ^*^
Motor score, mean (SD)	99.4 (11.1)	**97.8 (11.2)**	**101.4 (10.6)** ^*^
Cognitive score < 85, n (%)	26 (12.8)	17 (14.8)	9 (10.1)
Language score < 85, n (%)	59 (28.9)	38 (33.04)	21 (23.6)
Motor score < 85, n (%)	21 (10.29)	15 (13.04)	6 (6.7)
Cognitive or language or motor score < 85, n (%)	67 (32.84)	43 (37.4)	24 (27)

Note: ^*^p < 0.05 for comparisons between males and females. ^a^Magnetic resonance imaging or ultrasound scan was available for 141 infants (76 males and 65 females). Abbreviations: CBF_i_, cerebral blood flow index; CDO_2i_, cerebral oxygen delivery index; CMRO_2i_, cerebral metabolic rate of oxygen consumption index; HGB, hemoglobin concentration; IQR, 1 st and 3rd quartiles; OEF, cerebral oxygen extraction fraction; PMA, postmenstrual age; SD, standard deviation; SO_2_, cerebral oxygen hemoglobin saturation; TEA, term-equivalent age.

### Associations between neuromonitoring parameters and neurodevelopmental outcome

**Fig. 1:**
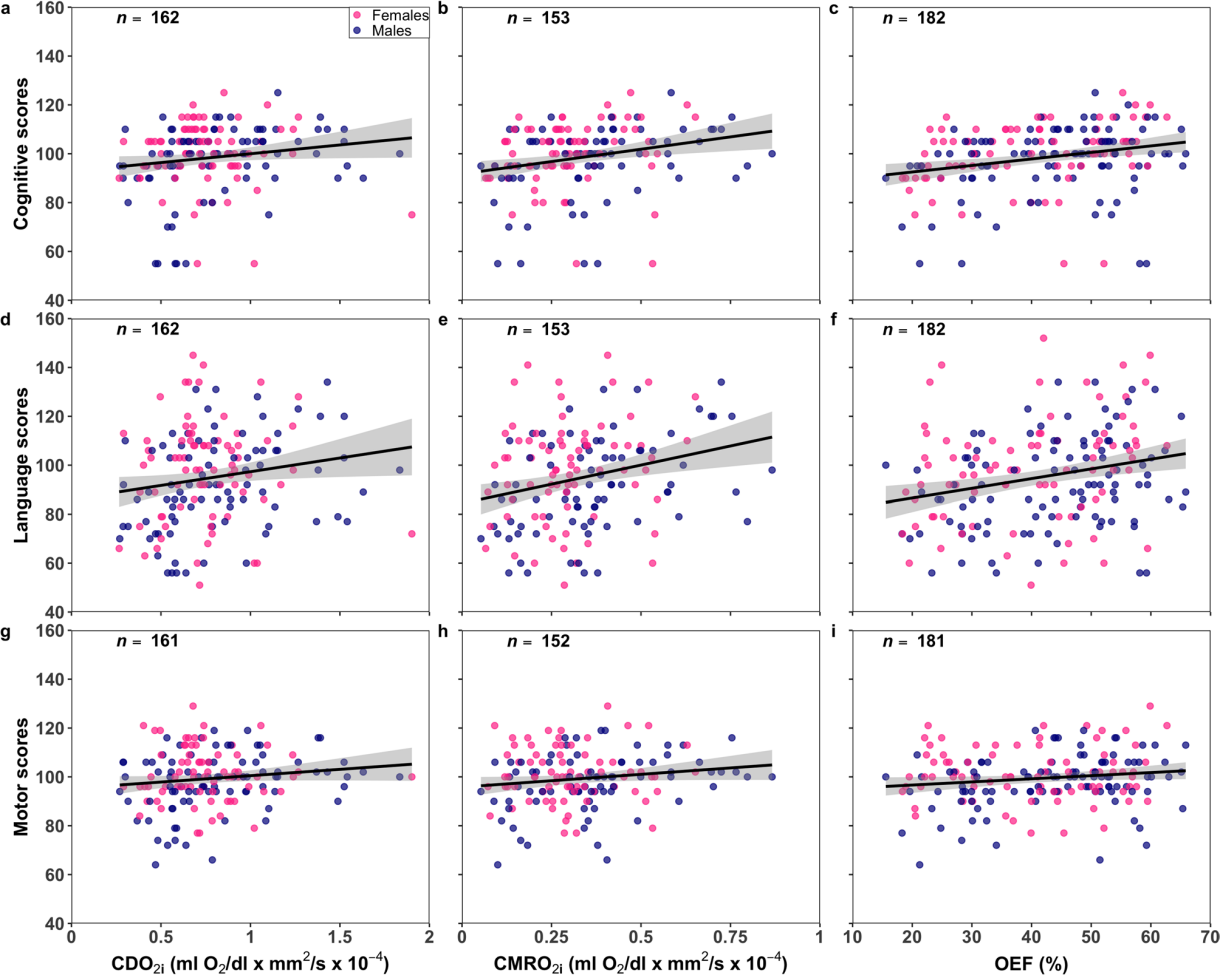
Neurodevelopmental outcomes (cognitive, language and motor) in function of bedside optical neuromonitoring parameters: (**a**, **d**, **g**) cerebral oxygen delivery index (CDO_2i_), (**b**, **e**, **h**) cerebral oxygen metabolism index (CMRO_2i_) and (**c**, **f**, **i**) cerebral oxygen extraction fraction (OEF). The sample size (*n*) is provided for each association. For visualization purpose, a linear fit (black line) with 95% confidence intervals (gray regions) are superimposed while data points are grouped by infant sex: males (blue) and females (pink).

Among all infants, CDO_2i_, CMRO_2i_ and OEF were positively associated with cognitive and language scores while SO_2_ was negatively associated (*R*^2^=[0.02–0.06], Fig. 1; Table 2). None of the associations between CBF_i_ and 2-year outcomes were significant. When adjusting for PMA, head circumference and socioeconomic status as confounders and including sex as an interaction term, all neuromonitoring parameters were associated with every neurodevelopmental domain (except for SO_2_ and OEF with motor scores; Table 2). These adjusted models explained up to 13% of the variance.

**Table 2:** Linear regression analyses between bedside optical neuromonitoring parameters measured around term-equivalent age and 2-year neurodevelopmental outcomes. For each neuromonitoring parameter, a second statistical model was adjusted for postmenstrual age at the time of neuromonitoring, head circumference and socioeconomic status as well as for sex as an interaction term.

	Cognitive			Language			Motor		
	β	CI	*R* ^2^	β	CI	*R* ^2^	β	CI	*R* ^2^
CBF_i_	8.88	[−4.2, 21.9]	0.01	18.5	[−0.1, 37.1]	0.02	4.3	[−6.6, 15.2]	−0.01
PMA	0.85	[−0.6, 2.3]	**0.11** ^†^	1.25	[−0.8, 3.3]	**0.13** ^†^	−0.07	[−1.3, 1.2]	**0.05** ^*^
HC	0.74	[−0.3, 1.8]		0.62	[−0.8, 2.1]		0.47	[−0.4, 1.3]	
SES	−12.0^†^	[−18.6, −5.4]		−14.7^**^	[−24.1, −5.4]		−7.34^*^	[−12.9, −1.6]	
CBF_i_: Sex	31.2^*^	[5.7, 56.8]		52.1^**^	[15.9, 88.2]		17.1	[−4.5, 38.7]	
CDO_2i_	7.2	[0.2, 14.2]	**0.02** ^*^	11.2^*^	[1.2, 21.2]	**0.03** ^*^	5.2	[−0.7, 11.1]	0.08
PMA	1.1	[−0.4, 2.5]	**0.13** ^†^	1.7	[−0.4, 3.8]	**0.11** ^†^	−0.05	[−1.3, 1.2]	**0.06** ^*^
HC	0.57	[−0.5, 1.6]		0.39	[−1.1, 1.9]		0.33	[−0.5, 1.2]	
SES	−11.9^†^	[−18.5, −5.4]		−14.4^**^	[−23.9, −5]		−7.27^*^	[−12.8, −1.7]	
CDO_2i_: Sex	18.9^*^	[4, 33.7]		21	[−0.4, 42.3]		7.89	[−4.7, 20.4]	
CMRO_2i_	20.2^**^	[7.3, 33.1]	**0.05** ^**^	31.3^**^	[12.5, 50]	**0.06** ^**^	10.5	[−0.6, 21.5]	0.02
PMA	0.5	[−1, 2]	**0.09** ^**^	1.41	[−0.7, 3.5]	**0.13** ^†^	−0.15	[−1.4, 1.1]	**0.05** ^*^
HC	0.09	[−1, 1.2]		0.09	[−1.5, 1.7]		0.11	[−0.8, 1]	
SES	−9.6^**^	[−16.7, −2.5]		−13.2^*^	[−23.2, −3.1]		−5.49	[−11.4, 0.4]	
CMRO_2i_: Sex	12.4	[−16.4, 41.2]		28.9	[−11.9, 69.7]		15.7	[−8.3, 39.7]	
OEF	26.7^†^	[11.5, 41.9]	**0.06** ^†^	39.6†	[16.8, 62.3]	**0.06** ^†^	12.8^*^	[0, 25.5]	0.02
PMA	0.88	[−0.4, 2.2]	**0.07** ^**^	1.93^*^	[0, 3.8]	**0.10** ^†^	−0.08	[−1.1, 1]	0.04
HC	0.28	[−0.7, 1.3]		0.61	[−0.9, 2.1]		0.2	[−0.6, 1]	
SES	−5.16	[−11.3, 1]		−7.84	[−16.9, 1.2]		−3.24	[−8.3, 1.8]	
OEF: Sex	−5.11	[−36.9, 26.6]		−7.04	[−53.5, 39.5]		13.9	[−12, 39.9]	
SO_2_	−0.37^†^	[−0.6, −0.2]	**0.06** ^†^	−0.57†	[−0.9, −0.3]	**0.06** ^†^	−0.17	[−0.3, 0]	0.06
PMA	0.88	[−0.4, 2.2]	**0.08** ^**^	1.89^*^	[0, 3.8]	**0.11** ^†^	−0.08	[−1.1, 1]	0.03
HC	0.25	[−0.8, 1.3]		0.54	[−0.9, 2]		0.19	[−0.6, 1]	
SES	−5.01	[−11.2, 1.2]		−7.33	[−16.3, 1.7]		−3.21	[−8.3, 1.8]	
SO_2_: Sex	0.05	[−0.4, 0.5]		0.08	[−0.6, 0.7]		−0.19	[−0.5, 0.2]	

Note: Statistical parameters are regression coefficient (*β*), 95% confidence intervals (CI) and adjusted model fit (*R*^*2*^). Abbreviations: CBF_i_, cerebral blood flow index; CDO_2i_, cerebral oxygen delivery index; CMRO_2i_, cerebral metabolic rate of oxygen consumption index; HC, head circumference; OEF, cerebral oxygen extraction fraction; PMA, postmenstrual age at the time of neuromonitoring; SES, socioeconomic status; SO_2_, cerebral oxygen hemoglobin saturation. ^*^p < 0.05, ^**^p < 0.01, ^†^*p* < 0.001.

Stratification by sex revealed associations between all neuromonitoring parameters and neurodevelopmental outcomes in males: CBF_i_, CDO_2i_, CMRO_2i_ and OEF were positively associated with every outcome (except for CBF_i_ with motor), while SO_2_ was negatively associated with every outcome (Table 3). Adjusted models in males explained up to 24% of the variance. In females, SO_2_ was negatively associated with cognitive and language scores while OEF was positively associated, providing 6–8% of the variance explained (Table 4). Adjusted models in females were not statistically significant.

**Table 3:** Linear regression analyses between bedside optical neuromonitoring parameters measured around term-equivalent age and 2-year neurodevelopmental outcomes in males. For each neuromonitoring parameter, a second statistical model was adjusted for postmenstrual age at the time of neuromonitoring, head circumference and socioeconomic status.

	Cognitive			Language			Motor		
	β	CI	*R* ^2^	β	CI	*R* ^2^	β	CI	*R* ^2^
CBF_i_	24.4^*^	[5, 43.7]	**0.06** ^*^	43.8^†^	[2.9, 13]	**0.12** ^†^	14	[−1.9, 30]	0.02
CBF_i_	20^*^	[0.29, 39.7]	**0.22** ^‡^	39.8^**^	[13.9, 65.7]	**0.21** ^†^	15	[−2.1, 32]	**0.09** ^*^
PMA	1.3	[−0.9, 3.6]		1.33	[−1.6, 4.3]		−0.44	[−2.4, 1.5]	
HC	1.19	[−0.1, 2.5]		0.87	[−0.8, 2.5]		0.79	[−0.3, 1.9]	
SES	−16.9^†^	[−26.3, −7.5]		−17.7^**^	[−30.1, −5.3]		−10.4^*^	[−18.5, −2.2]	
CDO_2i_	13.2^**^	[4.3, 22.1]	**0.08** ^**^	17.6^**^	[6.1, 29.2]	**0.09** ^**^	9^*^	[1.7, 16.3]	**0.06** ^*^
CDO_2i_	10.8	[2.1, 19.6]	**0.24** ^‡^	15.4^*^	[3.6, 27.1]	**0.18** ^†^	8.2^*^	[0.6, 15.8]	**0.11** ^*^
PMA	1.59	[−0.5, 3.7]		2.25	[−0.5, 5.1]		−0.25	[−2.1, 1.6]	
HC	0.87	[−0.4, 2.2]		0.44	[−1.3, 2.2]		0.53	[−0.6, 1.7]	
SES	−17.2^†^	[−26.5, −7.8]		−17.6^**^	[−30.2, −5]		−10.6^*^	[−18.7, −2.5]	
CMRO_2i_	25.6^**^	[8.9, 42.2]	**0.1** ^**^	42.9^†^	[21.4, 64.3]	**0.16** ^†^	19.3^**^	[5.8, 32.8]	**0.08** ^**^
CMRO_2i_	19.7^*^	[2, 37.4]	**0.18** ^†^	37^**^	[14, 60]	**0.23** ^†^	17.5^*^	[2.5, 32.4]	**0.09** ^*^
PMA	1.13	[−1.1, 3.4]		1.87	[−1, 4.8]		−0.54	[−2.4, 1.4]	
HC	0.35	[−1.1, 1.8]		−0.05	[−1.9, 1.8]		0.21	[−1, 1.4]	
SES	−15.9^**^	[−26.3, −5.4]		−17.6^*^	[−31.3, −4]		−8.35	[−17.2, 0.5]	
OEF	28.2^*^	[6.7, 49.8]	**0.05** ^*^	43.6^**^	[14.8, 72.4]	**0.07** ^**^	23.6^**^	[6.8, 40.4]	**0.06** ^**^
OEF	15.2	[−8.5, 38]	**0.09** ^*^	26.1	[−5.1, 57.4]	**0.13** ^**^	19.1^*^	[0.2, 38.1]	0.04
PMA	1.61	[−0.2, 3.5]		3.03^*^	[0.6, 5.5]		−0.03	[−1.5, 1.5]	
HC	0.72	[−0.6, 2]		0.53	[−1.2, 2.2]		0.42	[−0.6, 1.4]	
SES	−7.24	[−15.9, 1.4]		−9.35	[−20.7, 2.03]		−2.36	[−9.3, 4.5]	
SO_2_	−0.4^**^	[−0.7, −0.1]	**0.06** ^**^	−0.64^**^	[−1, −0.3]	**0.09** ^**^	−0.32^**^	[−0.6, −0.1]	**0.06** ^**^
SO_2_	−0.22	[−0.6, 0.1]	**0.09** ^*^	−0.4	[−0.8, 0.03]	**0.14** ^†^	−0.3	[−0.5, 0.01]	0.04
PMA	1.59	[−0.3, 3.5]		2.95^*^	[0.5, 5.4]		−0.03	[−1.5, 1.5]	
HC	0.69	[−0.6, 2]		0.45	[−1.2, 2.1]		0.4	[−0.6, 1.4]	
SES	−7.09	[−15.8, 1.6]		−8.86	[−20.2, 2.53]		−2.32	[−9.2, 4.6]	

Note: Statistical parameters are regression coefficient (*β*), 95% confidence intervals (CI) and adjusted model fit (*R*^*2*^). Abbreviations: CBF_i_, cerebral blood flow index; CDO_2i_, cerebral oxygen delivery index; CMRO_2i_, cerebral metabolic rate of oxygen consumption index; HC, head circumference; OEF, cerebral oxygen extraction fraction; PMA, postmenstrual age at the time of neuromonitoring; SES, socioeconomic status; SO_2_, cerebral oxygen hemoglobin saturation. ^*^p < 0.05, ^**^p < 0.01, ^†^*p* < 0.001, ^‡^*p* < 0.0001.

**Table 4:** Linear regression analyses between bedside optical neuromonitoring parameters measured around term-equivalent age and 2-year neurodevelopmental outcomes in females. For each neuromonitoring parameter, a second statistical model was adjusted for postmenstrual age at the time of neuromonitoring, head circumference and socioeconomic status.

	Cognitive			Language			Motor		
	β	CI	*R* ^2^	β	CI	*R* ^2^	β	CI	*R* ^2^
CBF_i_	−6.2	[−23.3, 10.9]	−0.01	−6.2	[−33.6, 21.1]	−0.01	−5.6	[−20.3, 9]	−0.01
CBF_i_	−10.3	[−28.8, 8.2]	−0.02	−12.7	[−41.8, 16.3]	−0.02	−5.3	[−20.8, 10.1]	−0.04
PMA	0.38	[−1.6, 2.3]		1.13	[−1.9, 4.2]		0.18	[−1.5, 1.8]	
HC	−0.6	[−2.4, 1.2]		−0.15	[−3, 2.7]		−0.5	[−2, 1]	
SES	−4.4	[−14.1, 5.4]		−10.3	[−25.7, 5.1]		−2.9	[−11.1, 5.3]	
CDO_2i_	−2.6	[−14.1, 9]	−0.01	2.6	[−15.8, 21.1]	−0.01	−0.6	[−10.7, 9.5]	−0.01
CDO_2i_	−5.5	[−18.4, 7.4]	−0.03	−3.6	[−23.9, 16.7]	−0.03	0.48	[−10.3, 11.3]	−0.04
PMA	0.43	[−1.6, 2.5]		1.05	[−2.1, 4.2]		0.05	[−1.6, 1.7]	
HC	−0.41	[−2.2, 1.4]		0.08	[−2.8, 2.9]		−0.4	[−1.9, 1.1]	
SES	−4.42	[−14.3, 5.4]		−10.2	[−25.7, 5.3]		−2.8	[−11, 5.5]	
CMRO_2i_	18.7	[−3.4, 40.8]	0.02	25.5	[−9.5, 60.5]	0.01	0.75	[−18.8, 20.3]	−0.01
CMRO_2i_	17.3	[−8.6, 43.1]	−0.01	13.9	[−26.4, 54.1]	−0.02	2.4	[−19.3, 24.1]	−0.04
PMA	−0.28	[−2.4, 1.8]		0.79	[−2.4, 4]		0.09	[−1.7, 1.8]	
HC	−0.62	[−2.5, 1.3]		0.15	[−2.8, 3.1]		−0.4	[−1.9, 1.2]	
SES	−2.48	[−12.7, 7.7]		−9.1	[−25, 6.8]		−2.7	[−11.2, 5.9]	
OEF	30.1^**^	[8.3, 51.9]	**0.08** ^**^	44.7^*^	[8.1, 81.4]	**0.06** ^*^	4.3	[−15.1, 23.8]	−0.01
OEF	28.9^*^	[5, 52.9]	0.04	40.1^*^	[0.4, 79.8]	0.03	5.05	[−15.5, 25.6]	−0.03
PMA	0.06	[−1.8, 1.9]		0.61	[−2.4, 3.6]		−0.1	[−1.6, 1.5]	
HC	−0.5	[−2.2, 1.2]		0.94	[−1.9, 3.8]		−0.2	[−1.6, 1.3]	
SES	−1.24	[−10.3, 7.9]		−5.35	[−20.4, 9.7]		−3.8	[−11.6, 4]	
SO_2_	−0.4^**^	[−0.7, −0.11]	**0.07** ^**^	−0.62^*^	[−1.1, −0.1]	**0.06** ^*^	−0.1	[−0.3, 0.2]	−0.01
SO_2_	−0.39^*^	[−0.7, −0.07]	0.04	−0.6^*^	[−1.1, −0.05]	0.04	−0.1	[−0.3, 0.2]	−0.03
PMA	0.09	[−1.7, 1.9]		0.63	[−2.4, 3.6]		−0	[−1.6, 1.5]	
HC	−0.5	[−2.2, 1.2]		0.92	[−1.9, 3.7]		−0.2	[−1.6, 1.3]	
SES	−1.19	[−10.3, 7.9]		−4.97	[−20, 10.1]		−3.8	[−11.6, 4]	

Note: Statistical parameters are regression coefficient (*β*), 95% confidence intervals (CI) and adjusted goodness of fit (*R*^*2*^). Abbreviations: CBF_i_, cerebral blood flow index; CDO_2i_, cerebral oxygen delivery index; CMRO_2i_, cerebral metabolic rate of oxygen consumption index; HC, head circumference; OEF, cerebral oxygen extraction fraction; PMA, postmenstrual age at the time of neuromonitoring; SES, socioeconomic status; SO_2_, cerebral oxygen hemoglobin saturation. ^*^p < 0.05, ^**^p < 0.01.

### Associations between postmenstrual age and neuromonitoring parameters

At the time of neuromonitoring, PMA was positively associated with CBF_i_, CDO_2i_, CMRO_2i_ and OEF while it was negatively associated with SO_2_ and HGB (*R*^2^=[0.02–0.09], Table 5). When adjusting regression models with head circumference and socioeconomic status as confounders and including sex as an interaction term with PMA, these associations explained up to 20% of the variance.

**Table 5:** Linear regression analyses between postmenstrual age at the time of bedside optical neuromonitoring and neuromonitoring parameters. Statistical models were also adjusted for head circumference and socioeconomic status as well as for sex as an interaction term. Statistical models were also performed when stratifying by sex and adjusted for head circumference and socioeconomic status.

	CBF_i_			CDO_2i_			CMRO_2i_		
	β	CI	*R* ^2^	β	CI	*R* ^2^	β	CI	*R* ^2^
**Full Cohort (n = 227)**									
PMA	0.03^†^	[0.01, 0.04]	**0.07** ^†^	0.04^**^	[0.01, 0.07]	**0.04** ^**^	0.02^**^	[0.01, 0.04]	**0.05** ^**^
HC	−0	[−0.02, 0.01]	**0.05** ^*^	0.02^*^	[0, 0.05]	**0.05** ^*^	0.02^**^	[0, 0.03]	**0.12** ^†^
SES	−0.02	[−0.09, 0.05]		−0.04	[−0.18, 0.09]		−0.08^*^	[−0.15, 0]	
PMA: Sex	0.02	[−0.02, 0.05]		−0.01	[−0.07, 0.04]		−0	[−0.04, 0.03]	
**Males (n = 126)**									
PMA	0.03^†^	[0, 1]	**0.11** ^†^	0.03	[−0.01, 0.08]	0.01	0.02	[0, 1]	0.02
HC	0.004	[−0.01, 0.02]	**0.08** ^*^	0.04^*^	[0, 0.1]	0.04	0.03^**^	[0.01, 0.04]	**0.09** ^*^
SES	0.003	[−0.09, 0.1]		−0	[−0.2, 0.2]		−0.06	[−0.2, 0.06]	
**Females (n = 101)**									
PMA	0.02^*^	[0, 0.04]	0.03	0.05^**^	[0.02, 0.08]	**0.10** ^**^	0.03^**^	[0.01, 0.04]	**0.09** ^**^
HC	−0.02	[−0.03, 0]	0.03	0.003	[−0.03, 0.03]	**0.06** ^*^	0.01	[−0.01, 0.02]	**0.09** ^*^
SES	−0.03	[−0.1, 0.1]		−0.06	[−0.22, 0.1]		−0.08	[−0.16, 0.01]	

	OEF			SO_2_			HGB		
	β	CI	*R* ^2^	β	CI	*R* ^2^	β	CI	*R* ^2^
**Full Cohort (n = 227)**									
PMA	0.01^*^	[0, 0.02]	**0.02** ^*^	−0.89^*^	[−1.7, −0.1]	**0.02** ^*^	−0.4^**^	[−0.7, −0.2]	**0.04** ^**^
HC	0.01^*^	[0, 0.02]	**0.11** ^‡^	−0.86^**^	[−1.5, −0.3]	**0.12** ^‡^	0.6^†^	[0.4, 0.8]	**0.20** ^‡^
SES	−0.09^†^	[−0.13, −0.04]		6.29^†^	[2.8, 9.8]		−0.22	[−1.4, 1]	
PMA: Sex	0.01	[−0.01, 0.03]		−0.75	[−2.3, 0.8]		−0.66^*^	[−1.2, −0.13]	
**Males (n = 126)**									
PMA	0.02^*^	[0, 0.03]	**0.04** ^*^	−1.27^*^	[−2.3, −0.2]	**0.04** ^*^	−0.7^†^	[−1.1, −0.3]	**0.09** ^†^
HC	0.01^**^	[0, 0.02]	**0.14** ^†^	−1.23^†^	[−1.9, −0.5]	**0.17** ^‡^	0.65^†^	[0.4, 0.9]	**0.30** ^‡^
SES	−0.09^**^	[−0.2, −0.03]		6.27^**^	[1.9, 10.7]		0.28	[−1.3, 1.8]	
**Females (n = 101)**									
PMA	0.01	[−0.01, 0.02]	−0	−0.47	[−1.7, 0.7]	−0	−0.14	[−0.5, 0.24]	−0.004
HC	0.003	[−0.01, 0.02]	0.02	−0.19	[−1.3, 0.9]	0.02	0.52^**^	[0.2, 0.8]	**0.07** ^*^
SES	−0.08^*^	[−0.16, 0]		6.06^*^	[0.4, 11.8]		−0.96	[−2.8, 0.9]	

Note: Statistical parameters are regression coefficient (*β*), 95% confidence intervals (CI) and adjusted goodness of fit (*R*^*2*^). Abbreviations: CBF_i_, cerebral blood flow index; CDO_2i_, cerebral oxygen delivery index; CMRO_2i_, cerebral metabolic rate of oxygen consumption index; HC, head circumference; HGB, blood hemoglobin concentration; OEF, cerebral oxygen extraction fraction; PMA, postmenstrual age at the time of neuromonitoring; SES, socioeconomic status; SO_2_, cerebral oxygen hemoglobin saturation. ^*^p < 0.05, ^**^p < 0.01, ^†^*p* < 0.001, ^‡^*p* < 0.0001.

When stratifying by sex, PMA was positively associated with CBF_i_ and OEF while it was negatively associated with SO_2_ and HGB in males (*R*^2^=[0.04–0.11], Table 5). When adjusted, these associations were stronger (except for CBF_i_) and explained up to 30% of the variance. PMA was also positively associated with CMRO_2i_ (*R*^2^ = 0.09). In females, PMA was positively associated with CDO_2i_ and CMRO_2i_ (*R*^2^=[0.09–0.10]) and provided similar *R*^*2*^ values when adjusting models (except for HGB; *R*^2^ = 0.07).

## Discussion

In this prospective observational cohort study of preterm infants born at 29–36 weeks’ GA, bedside optical neuromonitoring parameters measured around TEA were associated with 2-year neurodevelopmental outcome and PMA at the time of neuromonitoring. Increased cerebral oxygen availability (increased CDO_2i_) and utilization (increased CMRO_2i_ and OEF) were associated with higher cognitive and language scores at 2 years corrected age. These associations were stronger when adjusted and associations between CMRO_2i_, CBF_i_ and CDO_2i_ with motor scores became statistically significant. Increased PMA was also associated with increased cerebral oxygen availability and utilization, which were also stronger when adjusted (except for CBF_i_). These associations may reflect increased metabolic demand to support ongoing maturational processes involved in brain growth. In addition, most of these associations were stronger in males compared to females, suggesting sex-specific brain development mechanisms and a potential relationship with the higher brain vulnerability in preterm males. These results support the potential utility of bedside optical neuromonitoring to provide early biomarkers of brain health and maturation in preterm infants. To the best of our knowledge, this is the first study examining such associations in this population.

The third trimester is a critical period of brain development characterized by a dramatic increase in brain size and surface area, expansion of the neuronal circuitry and initiation of myelination^37,38^. As the brain grows and matures, neuronal activity increases, leading to a rise in metabolic demands reflected by increased cerebral blood flow and oxygen/nutrients utilization^39^. In this study, cerebral oxygen availability and utilization increased with increasing PMA. These results are consistent with previous neuromonitoring/neuroimaging studies reporting similar trends in preterm infants of various GA and conditions^9,31,35,40–46^. Our findings suggest that optical neuromonitoring can track cerebral changes associated with brain maturation and potentially detect altered brain development.

Bedside optical neuromonitoring techniques are safe and non-invasive, making them useful for early neurodevelopmental prognostication. While associations between FDNIRS-DCS parameters and long-term outcomes have not been previously reported in preterm infants born at 29–36 weeks’ GA, other studies have demonstrated the predictive value of conventional NIRS relative SO_2_, which can be compared with our results involving absolute SO_2_. In very (born at 28–32 weeks’ GA) and extremely preterm infants, higher duration of cerebral hypoxia (low relative SO_2_) measured early in age was negatively associated with long-term outcomes^28,47,48^. However, another study reported no association between the burden of cerebral hypoxia in extremely preterm infants and 2-year outcome^49^. In the current study, lower SO_2_ at TEA was associated with better outcome, which may not be consistent with some of these studies. This observation may be due to differences in several methodological aspects including GA range, timing of neuromonitoring, and neuromonitoring system. In addition, cerebral hypoxia is more commonly observed in very and extremely compared to moderate-to-late preterm (born at 32–36 weeks’ GA) infants. When measured alone, the interpretation of conventional NIRS relative SO_2_ is limited by physiological assumptions and can be confounded by blood flow and oxygen consumption^50^. In the current cohort, the decrease in SO_2_ with respect to PMA is likely primarily due to the transition from fetal to postnatal hemoglobin. This transition occurs in the first few months of life and is characterized by a large decrease in hematocrit, which affects hemoglobin concentrations and blood viscosity^51,52^. This observation was also reported in a previous study^31^. Our approach to measuring independently SO_2_ and CBF_i_ provided a more comprehensive and quantitative physiological assessment of brain health and maturation reflecting the interaction of individual biomarkers of cerebral oxygen availability and utilization.

MRI techniques can measure equivalent metrics to that of FDNIRS-DCS^53–55^. In very preterm infants, higher cerebral and cerebellar volumes and metabolism were associated with better outcomes^9,10,45,56^. In moderate-to-late preterm infants, white matter and cerebellar volumes at TEA were also positively associated with outcomes^19^. These MRI findings are likely consistent with ours and underscore the relevance of using neuromonitoring and neuroimaging parameters in the neonatal period as indicators of long-term neurodevelopmental outcome in preterm infants.

Sex was an important contributor to some associations between neuromonitoring parameters and PMA and neurodevelopmental outcome. This result is consistent with previous studies examining the influence of sex on brain development and long-term outcomes in preterm infants^3,11,19,21,23,24,45,56–58^. Stratification by sex further revealed stronger associations in males compared to females. Males had lower mean scores for all three neurodevelopmental domains (non-significant for cognition) compared to females. Male sex is a known risk factor for altered brain development and developmental delay in preterm infants^3,24,25^. Compared to females, males have larger brain volumes^11,59,60^, higher cerebral blood flow^36,61^ and lower relative SO_2_^62^. Total intracranial volume and brain parenchyma increased at a higher rate in males compared to females from 24 to 37 weeks’ GA^63^. Also, white matter microstructure was less organized in the motor area in males compared to females^11,16,24^. White matter alterations may persist through school age and affect cognitive function^21,64^. Our results suggest that neuromonitoring parameters are likely sensitive to brain vulnerability specific to males which may help orienting towards clinical phenotypes that warrant targeted surveillance and interventions.

However, the associations between CDO_2i_ and CMRO_2i_ with PMA were stronger in females. The stronger decrease in HGB levels with increasing PMA in males may have contributed to this observation as HGB is an important determinant of CDO_2i_ and CMRO_2i_. Higher data variability measured in CDO_2i_ and CMRO_2i_ in males may also have limited the statistical power of these associations.

Our study has several limitations. Previous studies have reported statistical models based on other confounding factors including gestational age, birth weight, ethnicity, brain injury on MRI as well as some MRI parameters^19,40,45,56^. In the current study, MRI was available for only a few patients and the other above clinical variables did not improve the performance of the models. The interpretation of our results is limited to the middle frontal cerebral physiology. However, middle, left and right frontal neuromonitoring parameters acquired in preterm infants yielded comparable findings in a previous study^35^. While most neuromonitoring sessions were performed in sleep state to minimize motion and poor data quality, measurements in wakeful state may have increased data variability. The TEA period is larger than that of the typical definition (37–42 weeks’ PMA) due to early and late discharge for some infants. At TEA, HGB was not always available due to early discharge and was estimated based on clinical charts^51^. The estimation of SO_2_ was based on an arterial/venous contribution measured in infants with congenital heart disease aged < 8 years^65^. However, previous neuromonitoring studies in preterm infants were also based on this assumption^31,35^. The neuromonitoring probe was originally designed for term newborns and its use in preterm infants with a smaller head may have decreased data quality due to improper head-probe contact. These low-quality data were flagged by our data rejection algorithm. Furthermore, skin pigmentation also affected FDNIRS signals and consequently sample sizes for SO_2_, OEF, and CMRO_2i_. This limitation could potentially be mitigated by signal calibration^66^. Inclusion criteria included a 48 h NICU admission as it is a known risk factor for developmental delay^67^. This methodological aspect could have resulted in selecting sicker neonates born between 29 and 36 weeks’ GA. Our findings are therefore applicable to this specific population.

In summary, bedside optical neuromonitoring parameters acquired in the NICU around TEA were associated with 2-year neurodevelopmental outcome and PMA in preterm infants born at 29–36 weeks of gestation. Increased cerebral oxygen availability and utilization were associated with increasing PMA and a better outcome at 2 years corrected age. These associations may indicate a rise in the metabolic demand to support brain growth and maturation. Additional or stronger associations with neurodevelopmental outcome were found when adjusting models. Most of these associations were stronger in males compared to females suggesting sex-specific brain development mechanisms and a potential relationship to the higher developmental vulnerability in males. These results suggest that bedside optical neuromonitoring may serve as a valuable tool to provide early biomarkers of brain health and maturation in preterm infants at TEA.

## Methods

### Study design and patients

In this single-centre prospective observational study, patients were consecutively recruited at the CHU Sainte-Justine University Hospital Center in Montreal (QC, Canada) from August 2018 to November 2021. Inclusion criteria included a diagnosis of premature birth between 29 and 36^6/7^ weeks’ GA and an admission to the NICU for at least 48 h after birth. Exclusion criteria included major chromosomal or congenital abnormality, neonatal stroke, hypoxic ischemic stroke, moribund infants, and infants under child protection services. For multiple births, the number of recruited siblings from the same pregnancy was limited to 10% of the study sample to reduce the impact of internal correlation within a pair. After this number was reached, one infant per multiple pregnancy was randomly selected. A previous study reported associations between bedside temporal changes in neuromonitoring parameters and neurological examination at TEA in a subset of this cohort^35^. The current study includes the full population with different specific objectives and analyses.

Parental written informed consent was obtained for each patient. This study (#2019 − 1936) was approved by the local institutional review board of the *Comité d’éthique de la recherche* at the *Centre de recherche Azrieli du CHU Sainte-Justine*, named by the Quebec Government (#FWA00021692) and acts in accordance with Quebec and Canada laws, and the Code of Federal Regulations in the USA. This study was conducted in accordance with the Declaration of Helsinki.

### Data sources and collection

Perinatal data were retrieved from medical charts. Demographic information was obtained from parental questionnaires. Socio-economic status was assessed using the guidelines of the *Institut de la Statistique du Québec*, which considers the total family income with respect to the total number of family members living in the household^68^. Peripheral arterial oxygen saturation (SpO_2_, %) was recorded from the clinical vital monitoring system during bedside optical neuromonitoring. HGB (g/dl) measured around the period of optical neuromonitoring was retrieved from medical charts. If not available due to early NICU discharge, HGB was estimated using published references based on PMA^51^. Neurological examination was performed at TEA using the Amiel-Tison and Gosselin Neurological Assessment^69^.

### Bedside optical neuromonitoring

Non-invasive neuromonitoring signals were acquired at the bedside using a commercial system (MetaOx, ISS Inc., Champaign, IL, USA) combining FDNIRS and DCS^70,71^. This system emits near infrared light at multiple wavelengths (from 690 to 852 nm) and collects photons after propagation through the neonatal head via optical fibers connected to a sensor. The optical sensor was optimally designed to collect light that has travelled through neonatal brain tissue^33,72^ and was previously used in other neonatal cohorts^35,73^. For FDNIRS, light sources and detectors were separated by 10, 15, 20, and 25 mm while for DCS, light was detected via eight optical fibres collocated at 22 mm from the source. Data acquisition was performed by positioning the sensor on the middle frontal position of the forehead. Quality assessments of FDNIRS and DCS signals were performed individually using published rejection criteria^33,35,41,74,75^. Data that were rejected due to low quality were not included for further analyses. Rejection criteria included data affected by motion, optical signals attenuated by hair or improper head-sensor coupling.

FDNIRS-DCS data were acquired around TEA, i.e. between 35 and 43 weeks of PMA, to assess cerebral physiology just before discharge when infant health was clinically stable and when neurological examination was performed. Each recording session lasted 25–30 min. To account for local inhomogeneities on the scalp and motion artifacts, FDNIRS-DCS signals were acquired up to 5 times. Energy exposure for FDNIRS-DCS laser sources was in accordance with the American National Standards Institute (ANSI) standard for safe use of lasers and related regulations^76^.

Data quality assessment and analysis were performed using MATLAB (Mathworks, Natick, MA, USA). Absolute concentrations of oxyhemoglobin (HbO_2_, µmol/dl) and deoxyhemoglobin (HbR, µmol/dl) were derived from FDNIRS signals and served to estimate SO_2_ (%) as the ratio of HbO_2_ and total hemoglobin (HbO_2_+ HbR). Tissue water concentration was assumed to be 85%^77^. Prior to FDNIRS data acquisition, data calibration was performed via measurement in a phantom designed with optical properties reflecting light-skin pigmentation. CBF_i_ (mm^2^/s) was derived from DCS signals^78,79^. OEF (%) was defined as the ratio between the arterial-venous oxygen saturation difference (SpO_2_ - SvO_2_) and SpO_2_, where SvO_2_ (%) was estimated through the use of SO_2_ and assuming an arterial: venous contribution ratio of 0.25:0.75^31,36,65,74,75,80^. CMRO_2i_ (ml O_2_/dl × mm^2^/s) was estimated using the Fick’s principle^81^, which was used in previous neonatal studies^31,36,74,75,80^. CMRO_2i_ was defined by the product of CBF_i_, HGB, OEF and the theoretical maximum oxygen carrying capacity ($\:\gamma\:$ = 1.39 ml O_2_/g of HGB). CDO_2i_ (ml O_2_/dl × mm^2^/s) was estimated by the product of $\:\gamma\:$, HGB, CBF_i_ and SpO_2_^82^. The sample sizes after applying rejection criteria were 204 for SO_2_ and OEF, 182 for CBF_i_ and CDO_2i_, and 169 for CMRO_2i_.

### Neurodevelopmental outcome

At 2 years corrected age, neurodevelopment was evaluated using the Bayley Scales of Infant and Toddler Development 4th Ed. (Bayley-4) by a trained occupational therapist blinded to neuromonitoring results. The Bayley-4 is a standardized norm-referenced assessment of cognitive, language and motor development (mean scores of 100 ± 15)^83^. Neurodevelopmental delay was defined as having at least one Bayley composite score < 85.

### Statistical analysis

Descriptive statistics were reported as mean and SD or median and IQR as well as frequencies and percentages for continuous and dichotomous data, respectively. Data normality was assessed for continuous variables by using a Q-Q plot and Kolmogorov-Smirnov test. Wilcoxon rank sum test and Fisher’s exact tests were performed to assess differences in continuous and dichotomous variables, respectively. Linear regression analysis was used to assess the associations between Bayley-4 scores and neuromonitoring parameters as well as between neuromonitoring parameters and PMA. Multiple linear regression analyses were also adjusted for potential confounding factors described in Table 1: antenatal corticosteroids, urgent cesarian section, gestational age, intrauterine growth restriction, birth weight, birth weight *z*-score, small for gestational age (birth weight < 10th percentile for the gestational age), head circumference at birth, sex, surfactant administration, respiratory support, PMA at the time of neuromonitoring, state (asleep or awake, or both) during neuromonitoring, neurological status at TEA, maternal education, and socioeconomic status. Other variables such as brain injury on ultrasound or MRI, retinopathy of prematurity, sepsis/meningitis and bronchopulmonary dysplasia were not frequently observed in our cohort of infants born at 29–36 weeks’ GA and therefore not used as statistical confounders. Statistical models were built by adding one confounder and/or interaction term at a time and were assessed with the model fit using adjusted *R*^2^. *P*-values lower than 0.05 were considered statistically significant. Statistical analyses were performed in RStudio (v4.1.3; R Core Team 2022)^84^.

## Data Availability

The datasets generated and/or analysed during the current study are available from the corresponding author on reasonable request.
